# Antioxidant and antidiabetic activity and phytoconstituents of lichen extracts with temperate and polar distribution

**DOI:** 10.3389/fphar.2023.1251856

**Published:** 2023-11-01

**Authors:** Alfredo Torres-Benítez, José Erick Ortega-Valencia, Nicolás Jara-Pinuer, Marta Sanchez, Gabriel Vargas-Arana, María Pilar Gómez-Serranillos, Mario J. Simirgiotis

**Affiliations:** ^1^ Instituto de Farmacia, Facultad de Ciencias, Universidad Austral de Chile, Valdivia, Chile; ^2^ Tecnológico Nacional de México, Instituto Tecnológico de Tlalnepantla, Tlalnepantla de Baz, Mexico; ^3^ Departamento de Farmacología, Farmacognosia y Botánica, Facultad de Farmacia, Universidad Complutense de Madrid, Madrid, Spain; ^4^ Laboratorio de Química de Productos Naturales, Instituto de Investigaciones de la Amazonía Peruana, Avenue Abelardo Quiñones, Iquitos, Peru; ^5^ Facultad de Industrias Alimentarias, Universidad Nacional de la Amazonía Peruana, Iquitos, Peru

**Keywords:** *Ochrolechia*, *Placopsis*, *Umbilicaria*, bioactive compounds, antioxidant, enzyme inhibition, Antarctic lichens

## Abstract

The objective of this research was to characterize the chemical composition of ethanolic extracts of the lichen species *Placopsis contortuplicata*, *Ochrolechia frigida*, and *Umbilicaria antarctica*, their antioxidant activity, and enzymatic inhibition through *in vitro* and molecular docking analysis. In total phenol content, FRAP, ORAC, and DPPH assays, the extracts showed significant antioxidant activity, and in *in vitro* assays for the inhibition of pancreatic lipase, *α*-glucosidase, and α-amylase enzymes, together with *in silico* studies for the prediction of pharmacokinetic properties, toxicity risks, and intermolecular interactions of compounds, the extracts evidenced inhibitory potential. A total of 13 compounds were identified by UHPLC-ESI-QTOF-MS in *P. contortuplicata*, 18 compounds in *O. frigida*, and 12 compounds in *U. antarctica*. This study contributes to the knowledge of the pool of bioactive compounds present in lichens of temperate and polar distribution and biological characteristics that increase interest in the discovery of natural products that offer alternatives for treatment studies of diseases related to oxidative stress and metabolic syndrome.

## 1 Introduction

Diabetes mellitus (DM), with a 3% age-standardized mortality rate, is one of the metabolic diseases that have increased in prevalence worldwide. It is characterized by the insufficient production of insulin by the pancreas or the ineffective degradation of the insulin produced (WHO, 2023). One of the pathophysiological mechanisms involved in the progression of diabetes is oxidative stress with the unbalanced generation of reactive oxygen species such as hydroxyl radical and superoxide anion and reduced activity of antioxidant mechanisms such as catalase, glutathione peroxidase, and superoxide dismutase (Mthiyane et al., 2022). Since ancient times and nowadays, natural products (plant, fungal, animal, microbial, or mineral) are being increasingly used compared to therapeutic alternatives due to the presence of active compounds with pharmacological properties (Cortes-Gallardo et al., 2004). Plants have been the most studied and used as resources with antidiabetic potential, with a report of more than 500 species, especially of the genera *Ficus, Artemisia, Solanum, Terminalia*, and *Euphorbia* (Salehi et al., 2019). The compounds that show the greatest benefit for the treatment of diabetes are polyphenols such as resveratrol, curcumin, quercetin, catechins, isoflavones, hydroxynamic acids, anthocyanins/anthocyanidins, kaempferol, and hesperidin, among others (Pandey and Rizvi, 2009; Naz et al., 2023).

**FIGURE 1:**
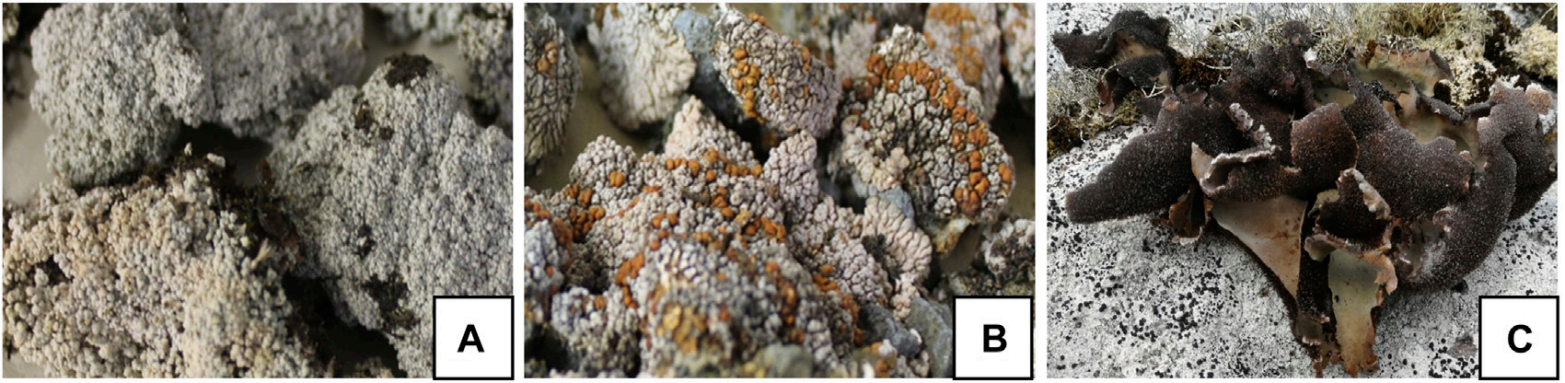
Lichen thallus of **(A)** *O. frigida*; **(B)** *P. contortuplicata*; and **(C)** *U. antarctica*.

**FIGURE 2:**
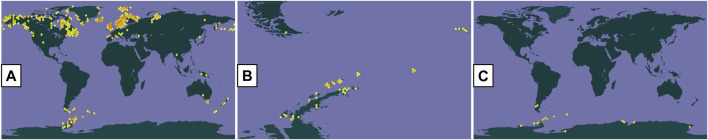
Distribution of lichen species (GBIF): **(A)** *O. frigida*; **(B)** *P. contortuplicata*; and **(C)** *U. antarctica*.

In this context, lichenized fungi or lichens are defined as an evolutionarily stable symbiosis between a fungus (mycobiont), algae (phycobiont), and/or cyanobacteria (cyanobiont) (Spribille et al., 2022), and they also represent a promising and efficient source of more than a thousand reported secondary metabolites with multiple biological activities (Poulsen-Silva et al., 2023) that are determined by biosynthetic gene clusters and are processed in the network of metabolic interactions of the primary symbionts, auxiliary symbionts, and microbiome that make up the lichen (Nazem-Bokaee et al., 2021; Singh, 2023). One of the groups of interest is the Antarctic lichen species, among which is *O. frigida*, characterized by a crustose, white thallus and lecanorine apothecia with a thin taline edge; *P. contortuplicata* with crustose thallus, marginal lobes separated by thin cracks or folded with a verrucous central part, and subspherical cephalodia generally orange–brown; and *U. antarctica* with foliose thallus attached to the substrate by a central umbilicus, gray-to-brown dorsal surface, black ventral surface with abundant simple ricines, and absent apothecia (Redón, 1985) (Figure 1). As for the growth substrate, *O. frigida* grows on mosses and plant debris, and *P. contortuplicata* and *U. antarctica* are species that grow on rocks, small stones, and nitrogen-rich soils [10]. These three species are distributed in the South Shetland Islands, South Orkney Islands, Antarctic Peninsula, and Antarctic circumpolar zone; in addition, *O. frigida* is also present in the Andes Mountain range in the southern area, and *P. contortuplicata* is considered a bipolar cosmopolitan species in the north and south latitudinal temperate zones (Seppelt, 1995) (Figure 2).

The objective of this research was to characterize the chemical composition of ethanolic extracts of the lichen species *P. contortuplicata, O. frigida*, and *U. antarctica*, their antioxidant activity, and enzymatic inhibition through *in vitro* and molecular docking analysis. The working hypothesis allows us to propose that the extracts of these Antarctic lichen species possess enzymatic inhibition activity, specifically for α-amylase, pancreatic lipase, and *α*-glucosidase, as well as antioxidative activity that would allow us to establish and guide efforts for the evaluation of a potential nutraceutical product for the treatment of metabolic diseases.

## 2 Materials and methods

### 2.1 Lichen material

A total of 100 g of the lichen species *O. frigida* (Sw.) Lynge (Ochrolechiaceae), *P. contortuplicata* I. M. Lamb (Trapeliaceae), and *U. antarctica* Frey & I. M. Lamb (Umbilicariaceae) was collected by A.T.-B., M.J.S., and N.J.-P. on Ardley Island, King George Island, South Shetland Archipelago (Maritime Antarctica), in February and December 2021. Specimens were determined by botanist Alfredo Torres-Benítez and deposited at the Natural Products Laboratory of the Universidad Austral (Valdivia, Chile), with identification numbers HL-01192021 (*O. frigida*), HL-01202021 (*P. contortuplicata*), and HL-01162021 (*U. antarctica*).

### 2.2 Preparation of ethanolic extracts

With each sample of lichen species, a total of 5 g was weighed and macerated using an analytical ethanol solvent three times, for 20 min per cycle, using ultrasound equipment at 35°C. Each extract solution was filtered and concentrated under reduced pressure at 38°C, obtaining three gummy extracts.

### 2.3 LC parameters and MS parameters

The analysis for the identification of bioactive compounds present in the lichen ethanolic extracts was performed on a UHPLC-ESI-QTOF-MS system equipped with UHPLC UltiMate 3000 RS using Chromeleon 6.8 software (Dionex GmbH, Idstein, Germany) and Bruker maXis ESI-QTOF-MS using the software Data Analysis 4.0 (all Bruker Daltonik GmbH, Bremen, Germany). 5 mg of each extract was dissolved in 2 mL of methanol for analysis and filtered using a polytetrafluoroethylene (PTFE) filter, and 10 μL of the extract was injected into the equipment. The chromatographic equipment consisted of a quaternary pump, an autosampler, a thermostated column compartment, and a photodiode array detector. Elution was performed using a binary gradient system with eluent (A) 0.1% formic acid in water and eluent (B) 0.1% formic acid in acetonitrile: 1% B isocratic (0–2 min), 1%–5% B (2, 3 min), 5% B isocratic (3–5 min), 5%–10% B (5–8 min), 10%–30% B (8–30 min), 30%–95% B (30–38 min), and 1% B isocratic (38–50 min). Separation was carried out using a Thermo 5 μm C18 80 Å column (150 mm × 4.6 mm) at a flow rate of 1.0 mL/min. ESI-QTOF-MS experiments were recorded in the negative ion mode, and the scan range was between 100 and 1,200 m/z. Electrospray ionization (ESI) conditions included a capillary temperature of 200°C, a capillary voltage of 2.0 kV, a dry gas flow rate of 8 L/min, and a nebulizer pressure of 2 bar, and the experiments were performed in an automatic MS/MS mode. The structural characterization of secondary metabolites was based on HR full MS, fragmentation patterns, and comparisons with the bibliography.

### 2.4 Total phenolic content

The content of total phenolic compounds was determined according to the Folin–Ciocalteu assay with each of the working solutions and was quantified spectrophotometrically based on a standard curve of gallic acid (Sánchez-Rangel et al., 2013). The analysis of the measurements was processed using a curve equation and coefficient of determination of y = 0.0606 x + 0.082 and *R* ^2^ = 0.9992.

### 2.5 Ferric-reducing antioxidant power assay

Trolox was used as standard and a stock concentration (1 mg/mL) diluted in ethanol was prepared, and the concentrations 125, 250, 500, and 1,000 μg/mL were obtained. Subsequently, 290 μL of working solution and 10 μL of each sample were mixed in a microplate well, and after 5 min, they were measured at 593 nm in a microplate reader (Benzie and Strain, 1996). The analysis of the measurements was processed with a curve equation and coefficient of determination of y = 0.0053 x + 0.0207 and *R* ^2^ = 0.9945.

### 2.6 Oxygen radical absorbance capacity assay

Trolox standard was prepared at the concentrations of 10, 20, 40, 40, 50, 60, 60, 80, 90, and 100 μM to make the calibration curve. Subsequently, 75 mM phosphate buffer at pH 7.0, fluorescein solution preincubated for 15 min at 37°C in microplate wells, compound 2,2′-azobis (2-amidinopropane) dihydrochloride (AAPH), and working solutions were used. The measurement was performed every 2 min for 30 min with excitation and emission wavelength at 485 and 530 nm, respectively (Cao and Prior, 1999). The analysis of the measurements was processed using a curve equation and coefficient of determination of y = 0.1337 x + 2.3241 and *R* ^2^ = 0.9890.

### 2.7 DPPH scavenging activity

A calibration curve of gallic acid was constructed to calculate its IC_50_ with a stock solution of 250 μg/mL gallic acid in absolute methanol and diluted to prepare solutions with the following concentrations: 1.525, 3.125, 6.225, 12.05, 25, 50, and 100 μg/mL. Subsequently, a methanolic solution of 400 μM DPPH was prepared, 50 μL of each sample was incorporated into the wells of the microplate, and 150 μL of 400 μM DPPH solution was added to each well, allowed to rest for 30 min, and measured at 515 nm in a microplate reader (Brand-Williams et al., 1995). The analysis of the measurements was processed with a curve equation and coefficient of determination of y = 2.5881 x + 6.5449 and *R* ^2^ = 0.9978.

### 2.8 *α*-Amylase inhibition assay

For each working solution, positive control and negative control in an Eppendorf tube of 150 µL of the sample, 200 µL of starch, 50 µL distilled water, and 100 µL of α-amylase enzyme were added and incubated for 5 min at 37°C. In another Eppendorf tube of 200 µL of the aforementioned mixture, 100 µL of DNS reagent was added and incubated for 20 min at 100°C. Subsequently, 900 µL of distilled water was added and cooled to room temperature. A measure of 200 μL of each solution was added to a microplate and measured at 515 nm using a microplate reader (Ali et al., 2006). The analysis of the measurements was processed with a curve equation and coefficient of determination of y = 0.2859 x + 35.186 and *R* ^2^ = 0.9602.

### 2.9 *α*-Glucosidase inhibition assay

The standard used in this assay was acarbose, with which a calibration curve was prepared using concentrations ranging from 250 to 0.025 μg/mL in 20 mM phosphate buffer (0.025, 0.125, 0.25, 12.5, 25, 62.5, 125, and 250 μg/mL). For ethanolic extracts, solutions at concentrations between 1,000 and 7.8 μg/mL (7.8, 15.6, 31.3, 62.5, 125, 250, 500, and 1,000 μg/mL) were prepared from a stock solution of 1 mg/mL in phosphate buffer (1,000 ug/mL). 50 μL of each of the working solutions were taken, and 50 μL of 5.0 mM pNPG was added and incubated for 5 min at 37°C. Subsequently, 100 μL of *α*-glucosidase enzyme (0.1 U/mL) was added, and absorbance was measured at 405 nm every 1 min for 20 min using a microplate reader (Liu et al., 2011). The analysis of the measurements was processed with a curve equation and coefficient of determination of y = 0.1963 x + 9.4416 and *R* ^2^ = 0.9485.

### 2.10 Pancreatic lipase inhibition assay

The standard used was orlistat, with which a calibration curve was prepared using concentrations ranging from 80 to 0.5 μg/mL in 70% ethanol (0.5, 1, 5, 10, 20, 30, 50, and 80 μg/mL). For ethanolic extracts, solutions were prepared at concentrations between 1,000 and 7.8 μg/mL (7.8, 15.6, 31.3, 62.5, 125, 250, 500, and 1,000) from a stock solution of 1 mg/mL in Tris-HCl buffer (1,000 μg/mL). With each working solution, 25 μL was taken with 50 μL of 5.0 mM NPC and 25 μL of pancreatic lipase enzyme (10 mg/mL) and incubated for 5 min at 37°C. Subsequently, measurement at 410 nm using a microplate reader was performed (Lewis and Liu, 2012). The analysis of the measurements was processed with a curve equation and coefficient of determination of y = 0.4871 x + 41.625 and *R* ^2^ = 0.9368.

### 2.11 Analysis of the pharmacological properties

The pharmacological properties of the phytochemicals identified by UHPLC-ESI-QTOF-MS obtained from the lichen species *O. frigida*, *P. contortuplicata*, and *U. antarctica* were evaluated; to determine whether the compounds obtained are favorable as inhibitors of α-amylase, α-glucosidase, and human pancreatic lipase, the pharmacokinetic properties were calculated using the Osiris DataWarrior (v 5.5.0) computational tool. Compounds were evaluated based on Lipinski’s rule, which states that an orally administered drug must have a molecular weight <500 Da, the partition coefficient (cLogP) must be <5, the number of bond donors of hydrogen must be <5, the number of hydrogen bond acceptors must be <10, and the number of spin bonds must be <10. The topological polar surface area and the percentage absorption (% ABS) (Eq. 1) were also calculated using the values calculated from TPSA in each of the compounds (Zhao et al., 2002; Ley-Martínez et al., 2022):

$$\%\text{ ABS}=109-\left(0.345\times \text{TPSA}\right).$$ (1)

### 2.12 Calculation of risk toxicity

To determine the toxicological behavior of the phytochemicals obtained from the lichen, the Osiris DataWarrior computational tool was used. The toxicological risks that were evaluated were mutagenicity, tumorigenicity, irritation, and reproductive effect (Ley-Martínez et al., 2022).

### 2.13 Molecular docking- ligand preparation

The two-dimensional structures of the phytochemicals that did not present any violation of Lipinsk’s relation as well as any risk of toxicity (2.5DHA, cyperine, diospyrol, hypoxyphenone, lecanoric acid, orsellinic acid, prephenic acid, SDA, O4BBA) were prepared using the ChemDraw 8.0 program (PerkinElmer Informatics, Waltham, MA, United States). Subsequently, the chemical structures of the ligands were imported into the Avogadro program (https://avogadro.cc, accessed on 06 June 2023) to optimize the geometry of the ligands using the MMFF94 force field function (Torres-Benítez et al., 2022; Torres-Benítez et al., 2023a). All the optimized compounds were saved in the mol2 format to carry out the molecular docking studies with the enzymes *α*-amylase, *α*-glucosidase, and human pancreatic lipase. Acarbose was used as the reference inhibitor for *α*-amylase and *α*-glucosidase enzymes (Swargiary and Daimari, 2020), while methoxy undecyl phosphonic acid (MUP) and orlistat were used as the reference inhibitors for the human pancreatic lipase enzyme (Trang et al., 2017).

Crystal structures of *α*-amylase (PDB:2QV4), *α*-glucosidase (maltase) (PDB: 2QMJ), and human pancreatic lipase (PDB:1LPB) were downloaded from the PDB database (http://www.rcsb.org/pdb). These crystal structures are crystallized with the acarbose ligand used as a reference inhibitor for *α*-amylase and *α*-glucosidase, while the MUP and orlistat ligands were used for human pancreatic lipase (Trang et al., 2017; Swargiary and Daimari, 2020). The information of the amino acids of the active site in the enzymes was used as a reference to carry out a targeted coupling in the catalytic sites in each of the crystallized inhibitors. Enzyme optimization was performed using UCSF Chimera software (v1.16, San Francisco, California, United States); water molecules were removed, and ligands were removed from the active site of crystallographic enzymes. All polar hydrogen atoms aggregated at pH = 7.4, considering the appropriate ionization states for basic and acidic amino acid residues (Silman et al., 1994; Torres-Benítez et al., 2022).

### 2.14 Docking simulation

After preparing the ligands (phytochemicals and acarbose) and the target proteins (*α*-amylase, *α*-glucosidase, and human pancreatic lipase), molecular docking was performed using the rigid crystalline enzyme structures and the flexible ligands where twist angles were identified (for 10 independent urns per ligand). Grid parameters (Table 1) were determined using the inhibitors crystallized in each of the enzymes as a reference (acarbose for *α*-amylase and *α*-glucosidase and MUP and orlistat for human pancreatic lipase). Each ligand was coupled with the enzymes separately, and the final energy (binding affinity in kcal/mol) of the ligand–enzyme interaction was obtained after each coupling. The evaluation of the interactions was carried out using the BIOVIA Discovery Studio program (v20.1.0.19295, San Diego: Dassault Systemes, 2020) (Torres-Benítez et al., 2022).

**TABLE 1:** Grid box parameters for docking α-amylase, α-glucosidase, and human pancreatic lipase.

Enzyme	Grid box size (Å)			Grid center coordinate		
	X	Y	Z	X	Y	Z
**α-Amylase**	50	50	50	12.37	48.13	26.24
**α-Glucosidase**	40	40	40	−20.83	−6.56	−5.04
**Human pancreatic lipase**	40	40	40	8.88	23.74	53.35

### 2.15 Statistical analysis

Three measurements were performed with each sample solution, and the results were expressed as mean values ±standard deviation using Microsoft Excel 2019 software. For comparison of means, a one-way analysis of variance (ANOVA with Tukey’s test at a significance level *p* ˂ 0.05) was calculated using GraphPad Prism 8 software.

## 3 Results and discussion

### 3.1 Qualitative analysis of phytoconstituents of lichen extracts

**FIGURE 3:**
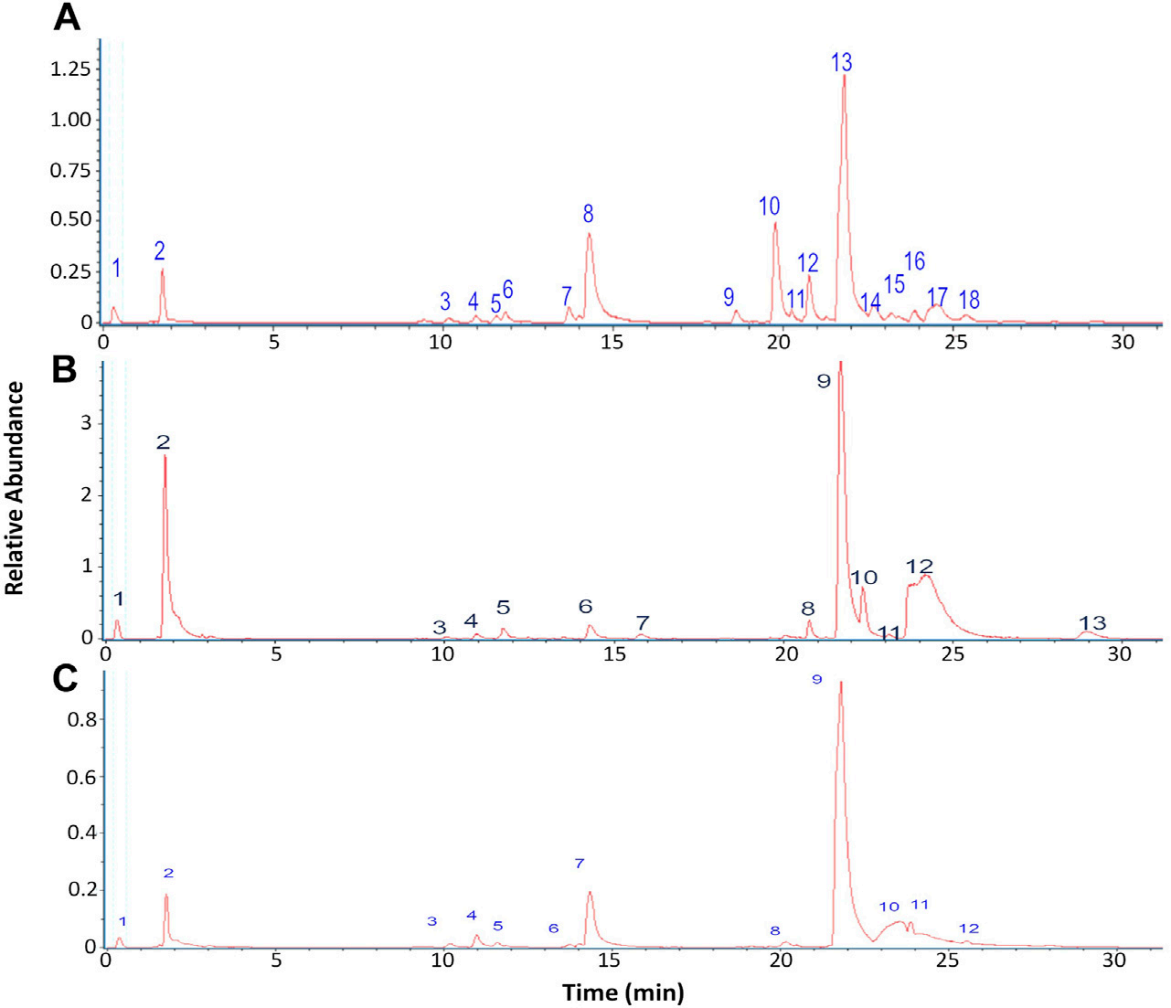
Chromatograms of lichen extracts: **(A)** *O. frigida*; **(B)** *P. contortuplicata*; and **(C)** *U. antarctica*.

**FIGURE 4:**
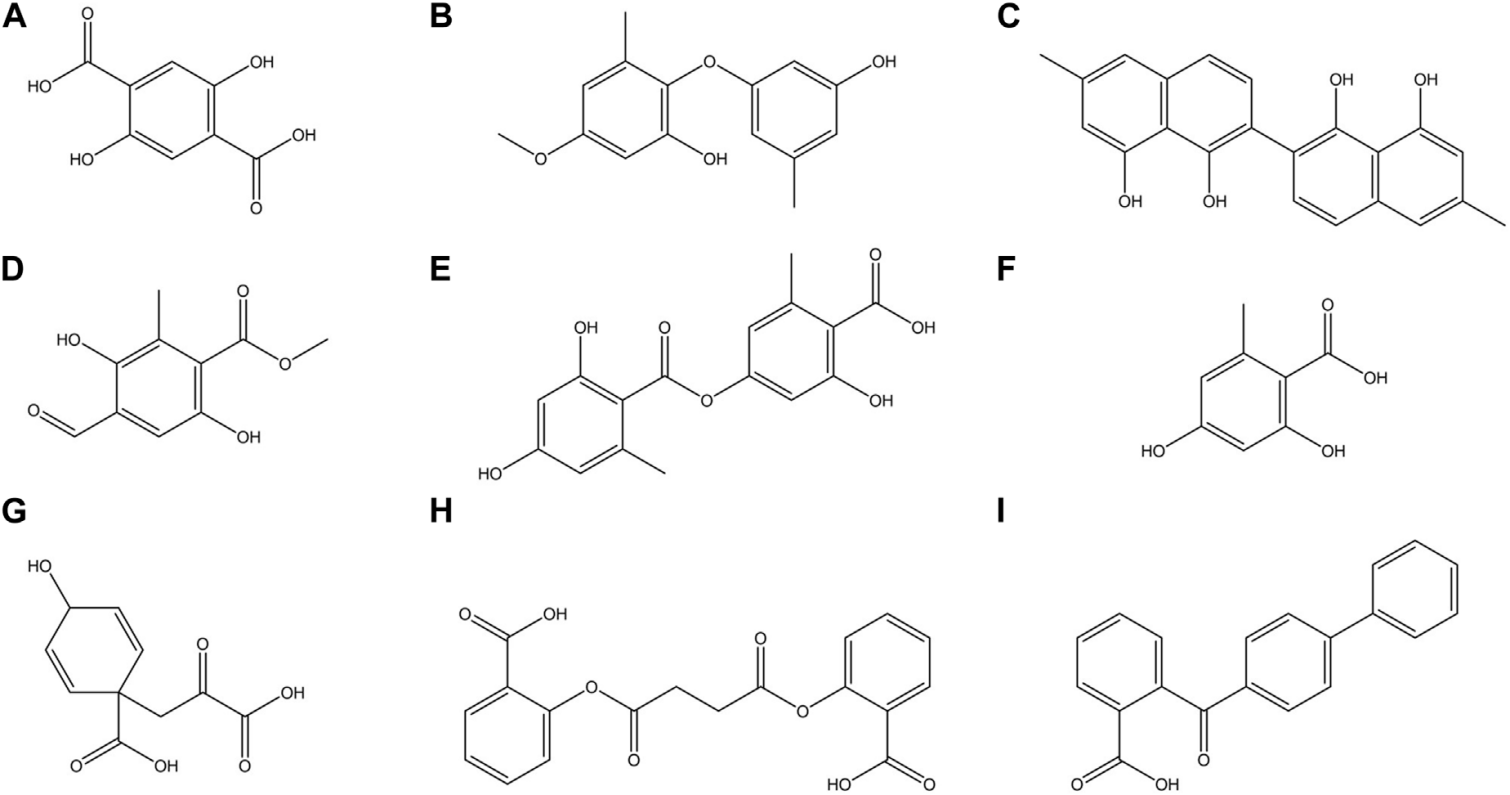
Chemical structures of some major compounds identified in ethanolic extracts of *O. frigida*, *P. contortuplicata*, and *U. antarctica*: **(A)** 2,5DHA (2,5-dihydroxyterephthalic acid); **(B)** cyperine; **(C)** diospyrol; **(D)** hypoxyphenone; **(E)** lecanoric acid; **(F)** orsellinic acid; **(G)** prephenic acid; **(H)** SDA (succinyldisalicylic acid); and **(I)** O4BBA (o-(4-biphenylylcarbonyl) benzoic acid).

The chromatographic analysis of the ethanolic extract of the lichen *O. frigida* by high-resolution mass spectrometric analysis (UHPLC-MS) in the negative mode allowed the identification of 18 compounds including aromatics, carbohydrates, acids, lipids, and depsides (Table 2; Figures 3, 4).

**TABLE 2:** Identification of bioactive compounds in *O. frigida* by UHPLC-ESI-QTOF-MS.

Peak	Tentative identification	[M-H]^-^	Retention time (min)	Theoretical mass (*m/z*)	Measured mass (*m/z*)	Accuracy (ppm)	Metabolite type	MS ions (ppm)
1	Na formate (internal standard)	C_4_H_2_O_4_	0.37	112.9829	112.9856	3.1	Ac	-
2	Mannitol	C_6_H_13_O_6_	1.83	181.0712	181.0723	3.9	C	151.0598
3	Citric acid	C_6_H_7_O_7_	10.04	191.0192	191.0184	4.2	Ac	111.0074
4	Azelaic acid	C_9_H_15_O_4_	11.02	187.0775	187.0969	−3.63	L	-
5	Hypoxyphenone	C_10_H_9_O_5_	11.22	209.0455	209.0415	8.8	A	167.0304
6	Prephenic acid	C_10_H_9_O_6_	11.82	225.0345	225.0365	−17.2	A	-
7	Orsellinic acid	C_13_H_9_O_5_	13.78	167.0314	167.0312	2.3	A	123.0432
8	3,4-Dihydroxybenzoic acid	C_7_H_5_O_4_	14.50	153.0169	153.0193	−15.6	A	109.0285
9	Undecyl glucoside	C_17_H_33_O_6_	18.68	333.2282	333.2225	−17.2	C	193.0056, 181.0059
10	Wedelolactone	C_16_H_9_O_7_	19.81	313.0295	313.0308	4.37	A	269.03821
11	Unknown	C_26_H_16_O_4_	20.05	392.1075	392.1054	5.32	d	350.0945
12	3,6,9,12-Tetraoxapentacosanoic acid	C_21_H_41_O_6_	20.80	389.2849	389.2853	0.87	L	315.2125
13	Lecanoric acid	C_16_H_13_O_7_	22.03	317.0666	317.0653	−3.2	d	167.0342
14	o-(4-Biphenylylcarbonyl) benzoic acid	C_20_H_13_O_3_	22.65	301.0870	301.0856	−4.5	A	167.0304
15	Tetradecyl glucoside	C_20_H_39_O_6_	23.25	375.2693	375.2681	−3.22	C	347.0362
16	18-Hydroxylinoleic acid	C_18_H_31_O_3_	24.21	295.2278	295.2279	−6.8	L	277.2133
17	18-Hydroxylinolenic acid	C_18_H_29_O_3_	24.54	293.2122	293.2071	−17.2	L	243.19740
18	Diospyrol	C_22_H_17_O_4_	25.28	345.1195	345.1132	−19.8	A	230.9846, 167.0309

A, aromatic; C, carbohydrate; Ac, acid; L, lipid; d, depside.

Acid derivatives: Peak 1 corresponded to Na formate (C_4_H_2_O_4_) used as the internal standard. Peak 3 was identified as citric acid (C_6_H_7_O_7_).

Carbohydrates: Peak 3 was identified as mannitol (C_6_H_13_O_6_), peak 9 as undecyl glucoside (C_17_H_33_O_6_) with diagnostic peaks at m/z 193.0056 and 181.0059, and peak 15 as tetradecyl glucoside (C_20_H_39_O_6_).

Lipids: Peak 4 was identified as azelaic acid (C_9_H_15_O_4_) and peak 12 as 3,6,9,12-tetraoxapentacosanoic acid (C_21_H_41_O_6_). Peaks 16 and 17 were identified as 18-hydroxy linoleic acid (C_18_H_31_O_3_) and 18-hydroxy linolenic acid (C_18_H_29_O_3_), respectively.

Aromatic derivatives: Peaks 5, 6, 7, 8, 10, and 14 were identified as hypoxyphenone (C_10_H_9_O_5_), prephenic acid (C_10_H_9_O_6_), orsellinic acid (C_13_H_9_O_5_), 3,4-dihydroxybenzoic acid (C_7_H_5_O_4_), wedelolactone (C_16_H_9_O_7_), and o-(4-biphenylylcarbonyl) benzoic acid (C_20_H_13_O_3_), respectively. Peak 18 was identified as diospyrol (C_22_H_17_O_4_) with diagnostic peaks at m/z 230.9846 and 167.0309.

Depsides: Peak 13 was identified as lecanoric acid (C_16_H_13_O_7_), and peak 11 was identified as an unknown compound (C_26_H_16_O_4_).

The chromatographic analysis of the ethanolic extract of the lichen *P. contortuplicata* by high-resolution mass spectrometric analysis (UHPLC-MS) in the negative mode allowed the identification of 13 compounds including aromatics, carbohydrates, acids, and depsides (Table 3; Figures 3, 4).

**TABLE 3:** Identification of bioactive compounds in *P. contortuplicata* by UHPLC-ESI-QTOF-MS.

Peak	Tentative identification	[M-H]^-^	Retention time (min)	Theoretical mass (*m/z*)	Measured mass (*m/z*)	Accuracy (ppm)	Metabolite type	MS ions (ppm)
1	Na formate (internal standard)	C_4_H_2_O_4_	0.37	112.9829	112.9856	3.1	Ac	-
2	Mannitol	C_6_H_13_O_6_	2.02	181.0712	181.0723	3.9	C	151.0598
3	3,4-Dihydroxybenzoic acid	C_7_H_5_O_4_	10.13	153.01953	153.02201	17.51	A	109.03217
4	Phthalic acid	C_8_H_5_O_4_	11.02	165.01933	165.02154	13.37	A	139.0379, 123.05044
5	3,4-Dihydroxybenzoic acid isomer	C_7_H_5_O_4_	11.76	153.0169	153.0193	−15.6	A	109.0285
6	Succinyldisalicylic acid	C_18_H_13_O_8_	14.31	357.06159	357.06131	−0.79	A	124.05044, 165.06600
7	Cyperine	C_15_H_15_O_4_	16.47	259.09758	259.10266	19.58	A	135.04781, 241.01395
8	Octadecyl beta-maltoside	C_30_H_57_O_11_	20.83	593.38985	593.39064	−1.32	A	389.29418, 259.00562, 241.0077
9	Lecanoric acid	C_16_H_13_O_7_	21.72	317.0666	317.0682	4.79	d	167.0378
10	Stearyl glucose	C_24_H_47_O_6_	22.34	431.3378	431.3404	6.01	C	289.0750, 149.0276
11	Crustinic acid	C_24_H_20_O_11_	23.15	483.0932	483.0963	6.31	A	431.3434, 333.03409
12	Lecanoric acid isomer	C_16_H_13_O_7_	24.17	317.0666	317.0681	4.71	d	167.03772
13	Tetrafucol A	C_24_H_17_O_12_	28.99	497.07255	497.07404	2.99	A	347.04615, 317.05815

A, aromatic; C, carbohydrate; Ac, acid; d, depside.

Acid derivatives: Peak 1 corresponded to Na formate (C_4_H_2_O_4_) used as the internal standard.

Carbohydrates: Peak 2 was identified as mannitol (C_6_H_13_O_6_), and peak 10 was identified as stearyl glucose (C_24_H_47_O_6_) with diagnostic peaks at m/z 289.0750 and 149.0276.

Aromatic derivatives: Peak 3 was identified as 3,4-dihydroxybenzoic acid (C_7_H_5_O_4_) ([M-H]- ion at m/z 153.01953 and diagnostic peak at m/z 109.03217), peak 4 as phthalic acid (C_8_H_5_O_4_) ([M-H]- ion at m/z 165.01933 and diagnostic peaks at m/z 139.0379 and 123.05044), and peak 5 as a 3,4-dihydroxybenzoic acid isomer (C_7_H_5_O_4_) ([M-H]- ion at m/z 153.0169 and diagnostic peak at m/z 109.0285). Peak 6, with a molecular anion at m/z 357.06159 and diagnostic peaks at m/z 124.05044 and 165.06600, was identified as succinyldisalicylic acid (C_18_H_13_O_8_), while peak 7 was identified as cyperine (C_15_H_15_O_4_) with an [M-H]- ion at m/z 259.09758 and diagnostic peaks at m/z 135.04781 and 241.01395 and peak 8 as octadecyl beta-maltoside (C_30_H_57_O_11_) with an [M-H]- ion at m/z 593.38985 and diagnostic peaks at m/z 389.29418, 259.00562, and 241.0077. Peak 11 was identified as crustinic acid (C_24_H_20_O_11_) with a molecular anion at m/z 483.0932 and diagnostic peaks at m/z 431.3434 and 333.03409. Peak 13, with a molecular anion at m/z 497.07255 and diagnostic peaks at m/z 347.04615 and 317.05815, was identified as tetrafucol A (C_24_H_17_O_12_).

Depsides: Peak 9 was identified as lecanoric acid (C_16_H_13_O_7_) and peak 12 as a lecanoric acid isomer with diagnostic peaks at m/z 167.0378 and 167.03772, respectively.

The chromatographic analysis of the ethanolic extract of the lichen *U. antarctica* by high-resolution mass spectrometric analysis (UHPLC-MS) in the negative mode allowed the identification of 12 compounds including aromatics, carbohydrates, acids, and depsides (Table 4; Figures 3, 4).

**TABLE 4:** Identification of bioactive compounds in *U. antarctica* by UHPLC-ESI-QTOF-MS.

Peak	Tentative identification	[M-H]^-^	Retention time (min)	Theoretical mass (*m/z*)	Measured mass (*m/z*)	Accuracy (ppm)	Metabolite type	MS ions (ppm)
1	Na formate (internal standard)	C_4_H_2_O_4_	0.37	112.9829	112.9856	3.1	Ac	-
2	Mannitol	C_6_H_13_O_6_	1.83	181.0712	181.0723	3.9	C	151.0598
3	Hypoxyphenone	C_10_H_9_O_5_	10.23	209.0455	209.0415	8.8	A	167.0304
4	Prephenic acid	C_10_H_9_O_6_	11.12	225.0345	225.0365	−17.2	A	-
5	2,5-dihydroxyterephthalic Acid	C_8_H_5_O_4_	11.72	197.0091	197.0064	−13.65	A	153.0181
6	Phthalic acid	C_8_H_5_O_4_	13.82	165.0193	165.0163	−18.27	A	139.0379
7	3,4-dihydroxybenzoic acid	C_7_H_5_O_4_	14.50	153.0169	153.0193	−15.6	A	109.0285
8	Orsellinic acid	C_8_H_7_O_4_	20.12	167.0349	167.0317	−19.22	A	124.0456
9	Lecanoric acid	C_16_H_13_O_7_	22.03	317.0666	317.0653	−3.2	d	167.0342
10	Rhein	C_15_H_8_O_6_	23.32	283.0189	283.0205	5.82	A	242.1724
11	Lecanoric acid isomer	C_16_H_13_O_7_	23.96	317.0666	317.0632	−10.95	d	167.0326
12	4-Hydroxyisophthalaldehyde	C_8_H_5_O_3_	25.28	149.0244	149.0230	−8.9	A	-

A, aromatic; C, carbohydrate; Ac, acid; d, depside.

Acid derivatives: Peak 1 corresponded to Na formate (C_4_H_2_O_4_) used as the internal standard.

Carbohydrates: Peak 2 was identified as mannitol (C_6_H_13_O_6_).

Aromatic derivatives: Peaks 3, 4, 5, 6, 7, 8, 10, and 12 were identified as hypoxyphenone (C_10_H_9_O_5_), prephenic acid (C_10_H_9_O_6_), 2,5-dihydroxyterephthalic acid (C_8_H_5_O_4_), phthalic acid (C_8_H_5_O_4_), 3,4-dihydroxybenzoic acid (C_7_H_5_O_4_), orsellinic acid (C_8_H_7_O_4_), rhein (C_15_H_8_O_6_), and 4-hydroxyisophthalaldehyde (C_8_H_5_O_3_), respectively.

Depsides: Peak 9 was identified as lecanoric acid (C_16_H_13_O_7_) and peak 11 as a lecanoric acid isomer with diagnostic peaks at m/z 167.0342 and 167.0326, respectively.

In recent years, metabolomic studies have intensified in lichenic species of tropical and, especially, temperate distribution and polar territories, strengthening the bank of reported compounds that make up chemotaxonomic variables for the differentiation of species and their complexes (Torres-Benítez et al., 2017). Likewise, the use of robust state-of-the-art techniques such as ultra-high performance liquid chromatography with a diode array (UHPLC-DAD) coupled to an electrospray ionization tandem mass spectrometer (ESI-MS-MS) traditionally used in plant extracts has also allowed greater precision in the identification and elucidation of bioactive compounds in lichens (Sepúlveda et al., 2022; Torres-Benítez et al., 2023b). Compounds present in the species *O. frigida*, *P. contortuplicata*, and *U. antarctica* are shared with other Antarctic species such as *Lecania brialmontii*, *Pseudephebe pubescens*, *Sphaerophorus globosus* (Torres-Benítez et al., 2022), *Cladonia gracilis*, *Cladonia chlorophaea* (Torres-Benítez et al., 2023a), and *Himantormia lugubris* (Areche et al., 2022), including carbohydrates, phenolic compounds (such as lecanoric acid, orselinic acid, and orcinol derivatives), and lipids.

The large number of compounds reported in lichens and classified in the groups of dibenzofurans, depsides, depsidones, depsones, lactones, quinones, and pulvinic acid derivatives are highly complex due to their synthesis through various biosynthetic pathways and their potential medicinal use as antibiotics, antitumor/antimutagenic, antiviral, enzyme inhibitor, and antioxidants (Bostie and Grube, 2005; Ureña-Vacas et al., 2022a; Ureña-Vacas et al., 2023). The lichen genera that group most species with reported biological activities are *Acarospora*, *Alectoria*, *Bryoria*, *Bulbothrix*, *Candelariella*, *Cetraria*, *Cetrelia*, *Cladia*, *Cladonia*, *Dirinaria*, *Evernia*, *Heterodermia*, *Hypogymnia*, *Lethariella*, *Lobaria*, *Melanelixia*, *Myelochroa*, *Parmelia*, *Parmotrema*, *Peltigera*, *Platismatia*, *Pleurosticta*, *Pseudevernia*, *Pseudoparmelia*, *Ramalina*, *Stereocaulon*, *Sticta*, *Teloschistes*, *Thamnolia*, *Umbilicaria*, *Usnea*, *Vulpicida*, and *Xanthoparmelia*; on the other hand, the isolated compounds with more biological evidence are atranorin, barbatic, diffractaic, evernic, fumarprotocetraric, gyrophoric, lobaric, orsellinic, physodic, protocetraric, usnic, and vulpinic acids (Adenubi et al., 2022).

### 3.2 Total phenolic content and antioxidant activity

Table 5 shows the values obtained from the total phenolic and antioxidant activity assays for the three ethanolic extracts under study. For phenolic content, the species *O. frigida* (1,000.6 ± 0.01 mg GAE) showed the highest values, followed by *P. contortuplicata* (561.2 ± 0.009 mg GAE) with medium values and *U. antarctica* with the lowest values (245 ± 0.011 mg GAE). In ORAC and FRAP, the results for the three extracts are comparable to the reported phenolic concentration, showing in *O. frigida* the optimum values 525.11 ± 0.135 and 45.004 ± 0.066 µmol Trolox/g, respectively. As for the DPPH analysis, the three extracts evidenced an IC_50_ value much higher than the gallic acid standard, suggesting a medium–low inhibition capacity of the study concentrations used; however, the extract of *O. frigida* continued to show better antioxidant activity than the extracts of *P. contortuplicata* and *U. antarctica* (307.981 ± 0.053, 441.106 ± 0.095, and 444.392 ± 0.066 μg/mL, respectively).

**TABLE 5:** Total phenolic content (TPC) and antioxidant activity (FRAP, ORAC, and DPPH) of the extracts of lichen species *O. frigida*, *P. contortuplicata*, and *U. antarctica*.

Assay	TPC (mg GAE/g)	FRAP (µmol Trolox/g)	ORAC (µmol Trolox/g)	DPPH IC_50_ (µg/mL)
*O. frigida*	1,000.6 ± 0.01^*^	45.004 ± 0.066^*^	525.11 ± 0.135^*^	307.981 ± 0.053^*^
*P. contortuplicata*	561.2 ± 0.009^*^	19.458 ± 0.027^*^	204.88 ± 0.632^*^	441.106 ± 0.095^*^
*U. antarctica*	245 ± 0.011^*^	8.367 ± 0.009^*^	82.77 ± 0.168^*^	444.392 ± 0.066^*^
Gallic acid^#^	-	-	-	2.24 ± 0.04^*^

The values represent the means ± SD of three replicates (*n* = 3). Values marked with * are statistically different using Tukey’s test at 0.05 level of significance (*p* ˂ 0.05). #, positive control.

These results correlate positively with the reports of extracts and compounds isolated from other lichen species that show highly effective phenolic concentration and antioxidant activity through colorimetric techniques (Luo et al., 2009; Mitrović et al., 2011; Jha et al., 2017; Studzińska-Sroka et al., 2021a; Elečko et al., 2022) and electrochemical, computational, and genetic studies (Kalra et al., 2023; Yañez et al., 2023). Likewise, the variability in the antioxidant properties of lichens is mediated by the geographical, altitudinal, and/or microhabitat conditions in which they develop working concentrations, metabolite isolation efficiency, types of solvent, and forms of extraction and exploration of the mechanisms of action (Ranković et al., 2012; White et al., 2014; Studzińska-Sroka et al., 2021b; Li et al., 2022; Popovici et al., 2022; Sánchez et al., 2022). On the other hand, the evident antioxidant capacity of lichenic species has supported their use for the evaluation of neuroprotective effects in *in vitro* and *in vivo* models, yielding positive results regarding cell viability, protection against induced oxidative stress, decrease in reactive oxygen species, improvement of mitochondrial function, and suppression of signaling pathways that induce inflammatory response in astrocytes (Fernández-Moriano et al., 2015; Fernández-Moriano et al., 2016; Lee et al., 2021; Ureña-Vacas et al., 2022b).

### 3.3 Enzymatic inhibitory activity

Table 6 shows the values obtained in the enzyme inhibition assays for the three extracts under study. For α-glucosidase, the extract of *O. frigida* presented the best activity (16 ± 0.015 μg/mL) with a lower IC_50_ compared to the standard acarbose; as for the extracts of *P. contortuplicata* and *U. antarctica*, the values obtained indicated a low inhibition of the enzyme (139.56 ± 0.056 and 151.94 ± 0.022 μg/mL, respectively). For pancreatic lipase, the three extracts showed low inhibition of the enzyme compared to the orlistat standard, with values similar to each other for *O. frigida* and *U. antarctica* (180.5535 ± 0.045 and 198.1632 ± 0.052 μg/mL, respectively) and an even higher value for *P. contortuplicata* (394.7333 ± 0.028 μg/mL). For α-amylase, the extracts show low inhibition of the enzyme compared to the standard orlistat; however, the best result was obtained in the extract of *P. contortuplicata* with an IC_50_ of 308.856 ± 0.036 μg/mL, followed by *U. antarctica* with an IC_50_ of 607.531 ± 0.038 μg/mL and *O. frigida* with the least efficient value of inhibition (1,609 ± 0.055 μg/mL).

**TABLE 6:** Enzyme inhibitory activity of the extracts of lichen species *O. frigida*, *P. contortuplicata*, and *U. antarctica*.

Assay	α-Glucosidase IC_50_ (µg/mL)	Pancreatic lipase IC_50_ (µg/mL)	α-Amylase IC_50_ (µg/mL)
*O. frigida*	16 ± 0.015^*^	180.5535 ± 0.045^*^	1,609.838 ± 0.055^*^
*P. contortuplicata*	139.56 ± 0.056^*^	394.7333 ± 0.028^*^	308.856 ± 0.036^*^
*U. antarctica*	151.94 ± 0.022^*^	198.1632 ± 0.052^*^	607.531 ± 0.038^*^
Orlistat^®#^	-	2.149 ± 0.008^*^	-
Acarbose^#^	206.614 ± 0.008^*^	-	6.477 ± 0.003^*^

The values represent the means ± SD of three replicates (*n* = 3). Values marked with * are statistically different using Tukey’s test at 0.05 level of significance (*p* ˂ 0.05). #, positive control.

The results obtained for the enzyme α-glucosidase with the three study species are comparable with those reported for the ethanolic extracts of the Antarctic species *C. gracilis* and *C. chlorophaea* (IC_50_ = 91.323 ± 0.010 and 108.590 ± 0.006 μg/mL, respectively), with less effective values against the acarbose standard, except for *O. frigida*, which showed the highest inhibitory potential (Torres-Benítez et al., 2023a). Similar values have been found in other species of *O. frigida*, such as *Xanthoria elegan*s (IC_50_ = >3 μg/mL), *X. parietina* (IC_50_ = 0.6 ± 0.0 μg/mL), *Parmotrema dilatatum* (IC_50_ = 17.5 μg/mL), and compounds isolated from *Parmotrema tsavoense* (IC_50_ = 10.7–17.6 μg/mL) (Devi et al., 2020; Duong et al., 2020; Mukemre et al., 2021). As for pancreatic lipase enzyme, *C. gracilis* and *C. chlorophaea* species (IC_50_ = 345.135 ± 0.050 and 125.310 ± 0.049 μg/mL, respectively) presented concentrations similar to those found in *O. frigida*, *P. contortuplicata*, and *U. antarctica*; however, in methanolic extracts of species such as *X. elegans*, *X. parietina*, and *Xanthoria candelaria* (IC_50_ = 79 ± 5, 68 ± 5 and 55 ± 3 μg/mL, respectively), better inhibition effectiveness was observed (Mukemre et al., 2021; Torres-Benítez et al., 2023a). For enzyme *α*-amylase, the reports in lichen species, especially those of temperate and polar distribution, are very scarce, and some species, such as *X. elegans*, *X. parietina*, and *X. candelaria* (IC_50_ = 2.1 ± 0.1, 1.7 ± 0.1, 2.0 ± 0.1 μg/mL, respectively), show effective values of enzyme suppression compared to those obtained in *P. contortuplicata*, *U. antarctica* and even greater difference in *O. frigida* (Mukemre et al., 2021).

### 3.4 Prediction of pharmacokinetic and toxicological properties

The pharmacokinetic properties of the phytochemicals obtained from the lichen species were evaluated using the Osiris DataWarrior computational tool (Table 7). For a compound to be considered as a potential orally administered drug, it must comply with Lipinski’s rules (Torres-Benítez et al., 2022; Torres-Benítez et al., 2023a). These rules allow the evaluation and monitoring of drugs according to their biological and pharmacological functions. The molecules that did not show any violation of Lipinski’s rules were 2.5DHA, 3.4DHA, 4-HSA, azelaic acid, cyperine, diospyrol, hypoxyphenone, lecanoric acid, orsellinic acid, phthalic acid, prephenic acid, rhein, SDA, wedelolactone, and O4BBA. As they do not present any violation of Lipinski’s rules, they can be considered as possible inhibitors of enzymes *α*-amylase, *α*-glucosidase, and human pancreatic lipase. In the same way, the toxicological analysis of all the compounds obtained from lichens was carried out using the Osiris DataWarrior computational tool (Torres-Benítez et al., 2022; Torres-Benítez et al., 2023a) where it was possible to observe that the compounds that did not present any risk of toxicity were 2.5DHA, cyperin, diospirol, hypoxyphenone, lecanoric acid, orselinic acid, prephenic acid, SDA, and O4BBA (Table 8). As they present no risk of toxicity and no violation of Lipinski’s rules, these compounds were proposed as possible inhibitors of enzymes *α*-amylase, *α*-glucosidase, and human pancreatic lipase. Therefore, they were evaluated by *in silico* analysis to observe their performance as inhibitors, comparing them with the known inhibitors of these enzymes (acarbose for α-amylase and α-glucosidase and MUP for human pancreatic lipase).

**TABLE 7:** Pharmacokinetic properties obtained from the software Osiris DataWarrior program of the phytochemicals obtained from lichens based on Lipinski’s rule.

Compound	%ABS^a^	TPSA (Å2)^b^	MW^c^	cLogP^d^	HBD^e^	HBA^f^	n-ROTB^g^	Violation of Lipinski’s rule
Rule	-	-	<500	≤5	≤5	≤10	≤10	≤1
18-HA	89.15	57.53	296.45	5.54	2	3	15	2
2,5DHA	69.30	115.06	198.13	−0.06	4	6	2	0
3,4DHA	82.17	77.76	154.12	0.45	3	4	1	0
TOPA	83.39	74.22	390.56	4.33	1	6	23	1
4-HSA	90.24	54.37	150.13	1.18	1	3	2	0
Azelaic acid	83.26	74.60	188.22	1.60	2	4	8	0
Crustinic acid	43.09	191.05	484.41	3.31	6	11	7	2
Cyperine	88.67	58.92	260.29	2.98	2	4	3	0
Diospyrol	81.08	80.92	346.38	5.01	4	4	1	0
Hypoxyphenone	80.08	83.83	210.18	1.16	2	5	3	0
Lecanoric acid	66.12	124.29	318.28	2.23	4	7	4	0
OBM	47.41	178.53	594.78	3.65	7	11	22	4
Orsellinic acid	82.17	77.76	168.15	0.80	3	4	1	0
Phthalic acid	83.26	74.60	166.13	0.63	2	4	2	0
Prephenic acid	70.39	111.9	226.18	−1.45	3	6	4	0
Rhein	70.39	111.9	284.22	1.83	3	6	1	0
SDA	65.12	127.2	358.30	2.19	2	8	9	0
Tetrafucol A	25.25	242.76	498.40	2.49	12	12	3	2
Wedelolactone	71.27	109.36	314.25	2.43	3	7	1	0
O4BBA	90.24	54.37	302.33	3.93	1	3	4	0

^a^ Percentage of absorption (%ABS).

^b^ Topological polar surface area (TPSA).

^c^ Molecular weight (MW).

^d^ Logarithm of partition coefficient between n-octanol and water (cLogP).

^e^ Number of hydrogen bond donors (HBD).

^f^ Number of hydrogen bond acceptors (HBA).

^g^ Number of rotable bonds (n-ROTB); 18HA, 18-hydroxylinoleic acid; 2,5DHA, 2,5-dihydroxyterephthalic acid; 3,4DHA, 3,4-dihydroxybenzoic acid; TOPA, 3,6,9,12-tetraoxapentacosanoic acid; 4-HAS, 4-hydroxyisophthalaldehyde; OBM, octadecyl beta-maltoside; SDA, succinyldisalicylic acid; O4BBA, o-(4-biphenylylcarbonyl) benzoic acid.

**TABLE 8:** Toxicity risk of the phytochemicals obtained from *O. frigida*, *P. contortuplicata*, and *U. antarctica* extracts.

Compound	Mutagenic	Tumorigenic	Reproductive effect	Irritant
18-HA	None	None	None	High
2,5DHA	None	None	None	None
3,4DHA	High	None	None	None
TOPA	None	None	None	High
4-HSA	None	None	None	High
Azelaic acid	None	None	None	High
Crustinic acid	None	None	None	None
Cyperine	None	None	None	None
Diospyrol	None	None	None	None
Hypoxyphenone	None	None	None	None
Lecanoric acid	None	None	None	None
OBM	None	None	None	None
Orsellinic acid	None	None	None	None
Phthalic acid	High	None	High	Low
Prephenic acid	None	None	None	None
Rhein	None	None	None	High
SDA	None	None	None	None
Tetrafucol A	None	None	None	None
Wedelolactone	None	None	High	None
O4BBA	None	None	None	None

18HA, 18-hydroxylinoleic acid; 2,5DHA, 2,5-dihydroxyterephthalic acid; 3,4DHA, 3,4-dihydroxybenzoic acid; TOPA, 3,6,9,12-tetraoxapentacosanoic acid; 4-HAS, 4-hydroxyisophthalaldehyde; OBM, octadecyl beta-maltoside; SDA, succinyldisalicylic acid; O4BBA, o-(4-biphenylylcarbonyl) benzoic acid.

The bioavailability of compounds present in lichens is assessed by topological polar surface area analysis (TPSA) (Torres-Benítez et al., 2022; Torres-Benítez et al., 2023a). This parameter is closely related to the passive molecular transport of drugs through cell membranes; it will allow predicting the behavior and properties of drug transport to assess their possible bioavailability. The TPSA parameter helped to predict the percentage absorption of the compounds (Torres-Benítez et al., 2022). The compounds that presented a higher percentage of absorption according to their TPSA values were 4-HSA (90.24%), O4BBA (90.24%), 18-HA (89.15%), cyperine (88, 67%), TOPA (83.39%), azelaic acid (83.26%), and phthalic acid (83.26%). With the results of the evaluation of the pharmacological and toxicological properties, it was observed that those compounds that did not present any toxicological risk and any violation of Lipinski’s rules were proposed as possible inhibitors of the enzymes to be evaluated; therefore, the compounds 2,5DHA, cyperin, diospyrol, hypoxyphenone, lecanoric acid, orsellinic acid, prephenic acid, SDA, and O4BBA were those that were used for the *in silico* analysis to observe the behavior against these enzymes, comparing them with known inhibitors such as acarbose, orlistat, and MUP (Trang et al., 2017; Swargiary and Daimari, 2020).

### 3.5 Evaluation of docking *α*-amylase inhibition

**FIGURE 5:**
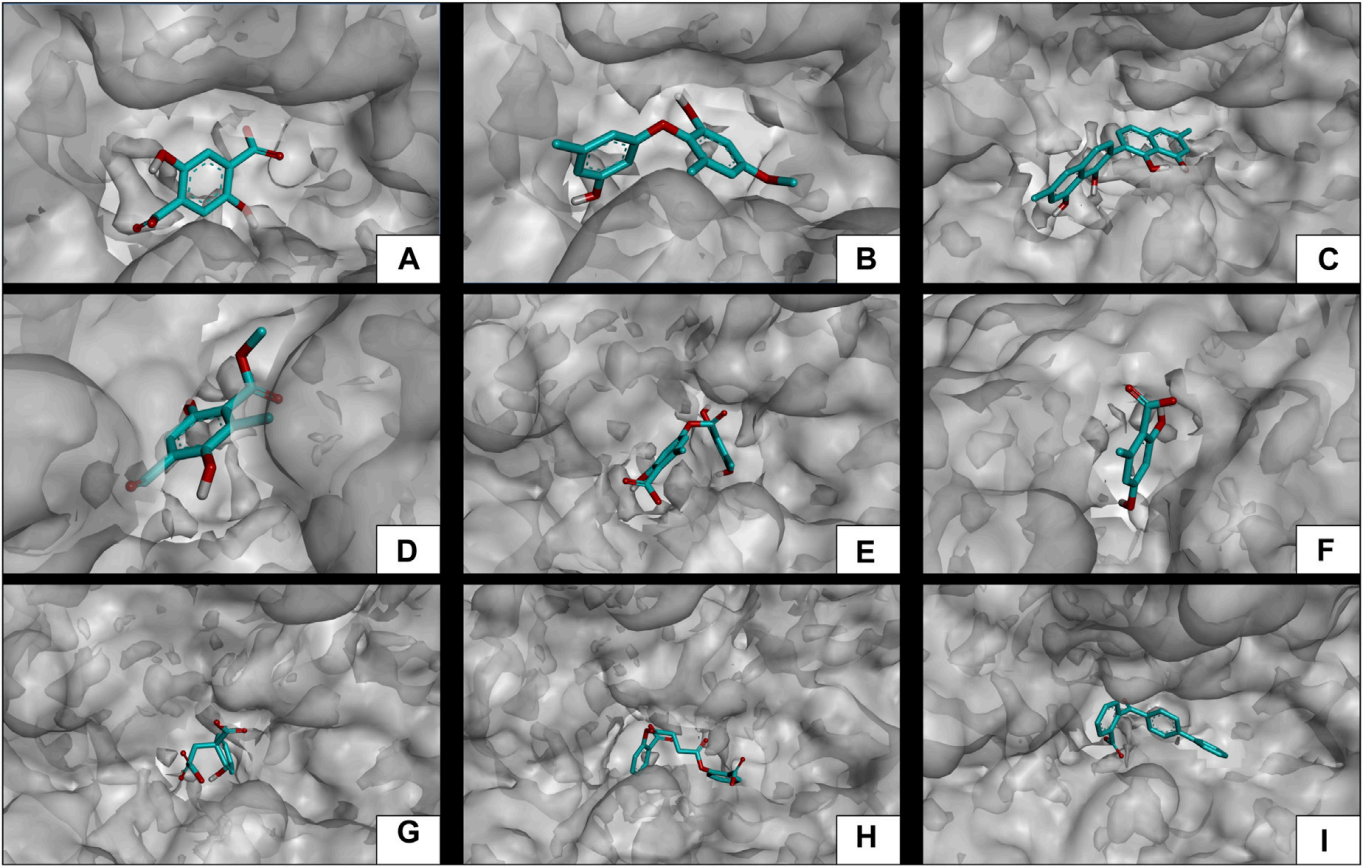
Docking molecular between phytochemicals and the α-amylase enzyme in a surface view. **(A)** *α*-Amylase and 2,5DHA; **(B)** *α*-amylase and cyperine; **(C)** *α*-amylase and diospyrol; **(D)** *α*-amylase and hypoxyphenone; **(E)** α-amylase and lecanoric acid; **(F)** *α*-amylase and orsellinic acid; **(G)** *α*-amylase and prephenic acid; **(H)** *α*-amylase and SDA; and **(I)** *α*-amylase and O4BBA.

**FIGURE 6:**
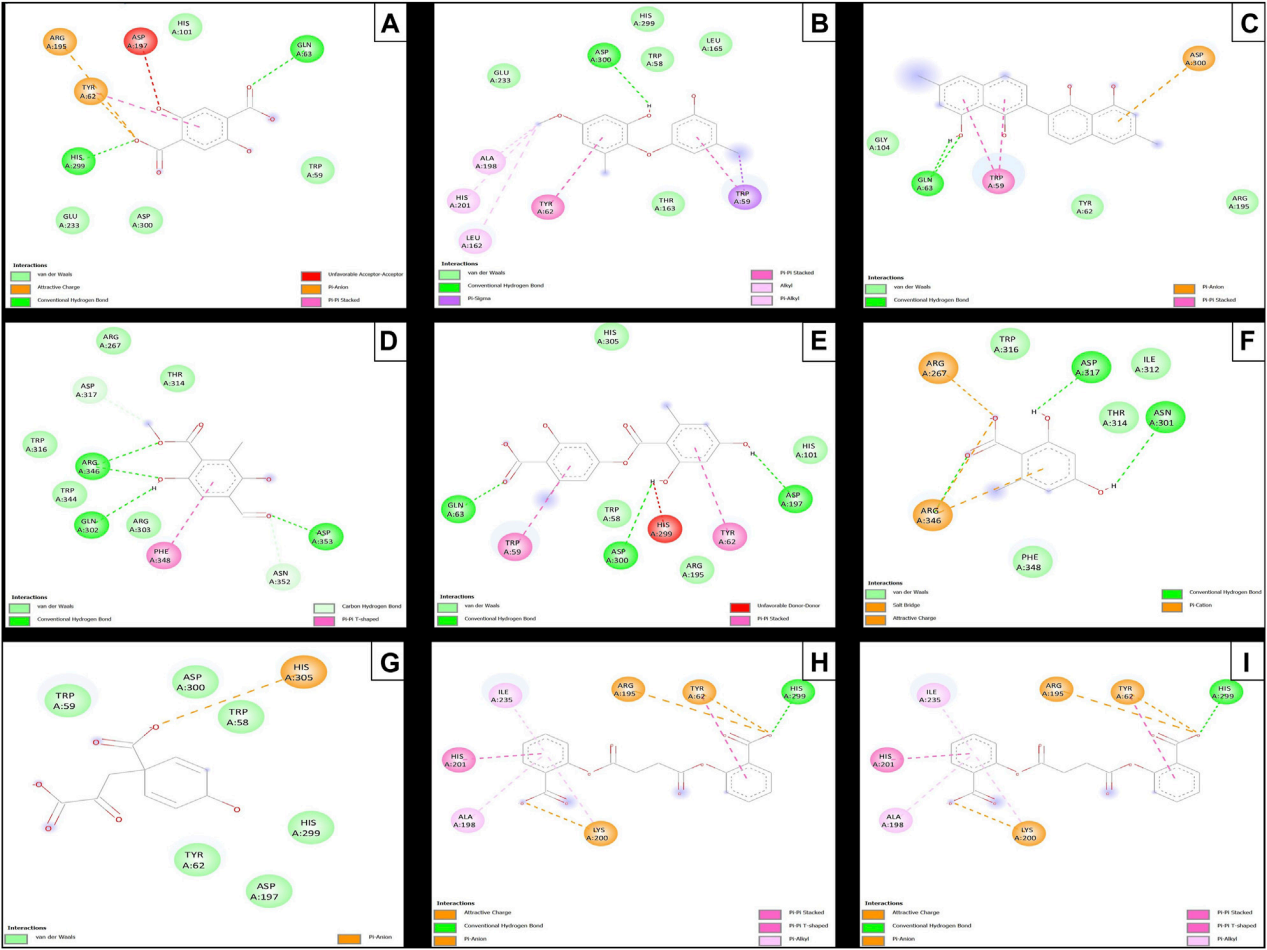
Molecular interactions of between phytochemicals and the *α*-amylase enzyme. **(A)** Molecular interactions of 2,5DHA and *α*-amylase; **(B)** molecular interactions of cyperine and *α*-amylase; **(C)** molecular interactions of diospyrol and *α*-amylase; **(D)** molecular interactions of hypoxyphenone and *α*-amylase; **(E)** molecular interactions of lecanoric acid and *α*-amylase; **(F)** molecular interactions of orsellinic and acid *α*-amylase; **(G)** molecular interactions of prephenic acid and *α*-amylase; **(H)** molecular interactions of SDA and *α*-amylase; and **(I)** molecular interactions of O4BBA and *α*-amylase.

Subsequent to the pharmacokinetic and toxicological analyses, those compounds that did not appear to be at any risk of toxicity, and no violation of Lipinski’s rules were chosen (Figure 4). These compounds (Figure 4) were proposed as potential α-amylase inhibitors and evaluated by *in silico* molecular sugar analysis to investigate their behavior and binding properties at the α-amylase catalytic site (Figures 5, 6). Table 9 shows the binding affinities of the compounds proposed as inhibitors and each of the enzymes. These compounds were compared with the reference inhibitor (acarbose) to see if the affinity at the catalytic site was similar or better. The study of the phytochemical–enzyme interaction showed that the compounds diospyrol, O4BBA, and lecanoric acid presented the highest binding affinities with the enzyme α-amylase. The binding affinities were found to be −9.00, −8.70, and −8.10 kcal/mol, respectively (Table 9). These affinities that these compounds appeared to have in comparison with the reference inhibitor acarbose (−7.80 kcal/mol) were higher because the map of ligand interactions (Figures 6C, E, I respectively) and the geometry adopted in the catalytic pocket (Figures 5C, E, I; respectively) were suitable for energetic and geometric stability at the catalytic site.

**TABLE 9:** Binding affinities of phytochemicals with α-amylase, α-glucosidase, and human pancreatic lipase enzymes.

Compound	α-Amylase (kcal/mol)	α-Glucosidase (kcal/mol)	Human pancreatic lipase (kcal/mol)
2,5DHA	−6.10	−5.80	−6.60
Cyperine	−7.30	−7.20	−8.10
Diospyrol	−9.00	−8.80	−11.0
Hypoxyphenone	−6.00	−4.90	−6.80
Lecanoric acid	−8.10	−7.00	−9.00
Orsellinic acid	−6.10	−5.10	−6.50
Prephenic acid	−6.10	−4.90	−6.50
SDA	−7.60	−7.30	−8.50
O4BBA	−8.70	−8.40	−10.5
Acarbose*	−7.80	−7.00	-
Orlistat*	-	-	−7.10
MUP*	-	-	−5.70

5DHA, 2,5-dihydroxyterephthalic acid; SDA, succinyldisalicylic acid; O4BBA, o-(4-biphenylylcarbonyl) benzoic acid; MUP, methoxy undecyl phosphonic acid. *, reference inhibitor.

The diospyrol compound presented two conventional H bonds with the amino acid Gln63 because the hydroxyl functional group of one of its aromatic rings allows the donation of a hydrogen bond as well as the acceptance of a hydrogen bond, and it is also observed that diospyrol presents a *π*-anion-type interaction between the *π* electrons of the aromatic ring and the amino acid Asp300. These interactions are highly involved in the energetic and geometric stability of the diospyrol compound, so this compound had a higher binding affinity at the α-amylase catalytic site (Figure 5C). The compound O4BBA and lecanoric acid showed similar values in the binding affinity (−8.70 and −8.10 kcal/mol, respectively); however, it is observed that lecanoric acid has a lower binding affinity because it presents an unfavorable donor–donor interaction between a hydroxyl group of one of its aromatic rings and the amino acid His299; however, it is observed that it presents a higher binding affinity than the reference inhibitor acarbose because it presents three strong H-bond interactions with the amino acids Gln63, Asp197, and Asp300 (Figure 6E). Compounds SDA and cyperine presented binding affinities (−7.60 and −7.30 kcal/mol, respectively) similar to those of the reference inhibitor acarbose (−7.80 kcal/mol) because the geometric distribution (Figures 5B, H) and the interactions at the catalytic site (Figures 6B, H, respectively) were very similar to those of acarbose. Both compounds presented a strong H-bond-type interaction; the SDA compound interacted with His299 amino acid (Figure 6H), while the cyperine compound interacted with Asp300 amino acid (Figure 6B).

The compounds 2.5DHA, hypoxyphenone, orselinic acid, and prephenic acid had similar and lower binding affinities (−6.10, −6.00, −6.10, and −6.10 kcal/mol, respectively) compared to the inhibitor acarbose; this is mainly because the binding was carried out of the catalytic site of *α*-amylase, so the geometric distribution in the binding site was not the most adequate (Figures 5A, D, F, G; respectively).

### 3.6 Evaluation of docking *α*-glucosidase inhibition

**FIGURE 7:**
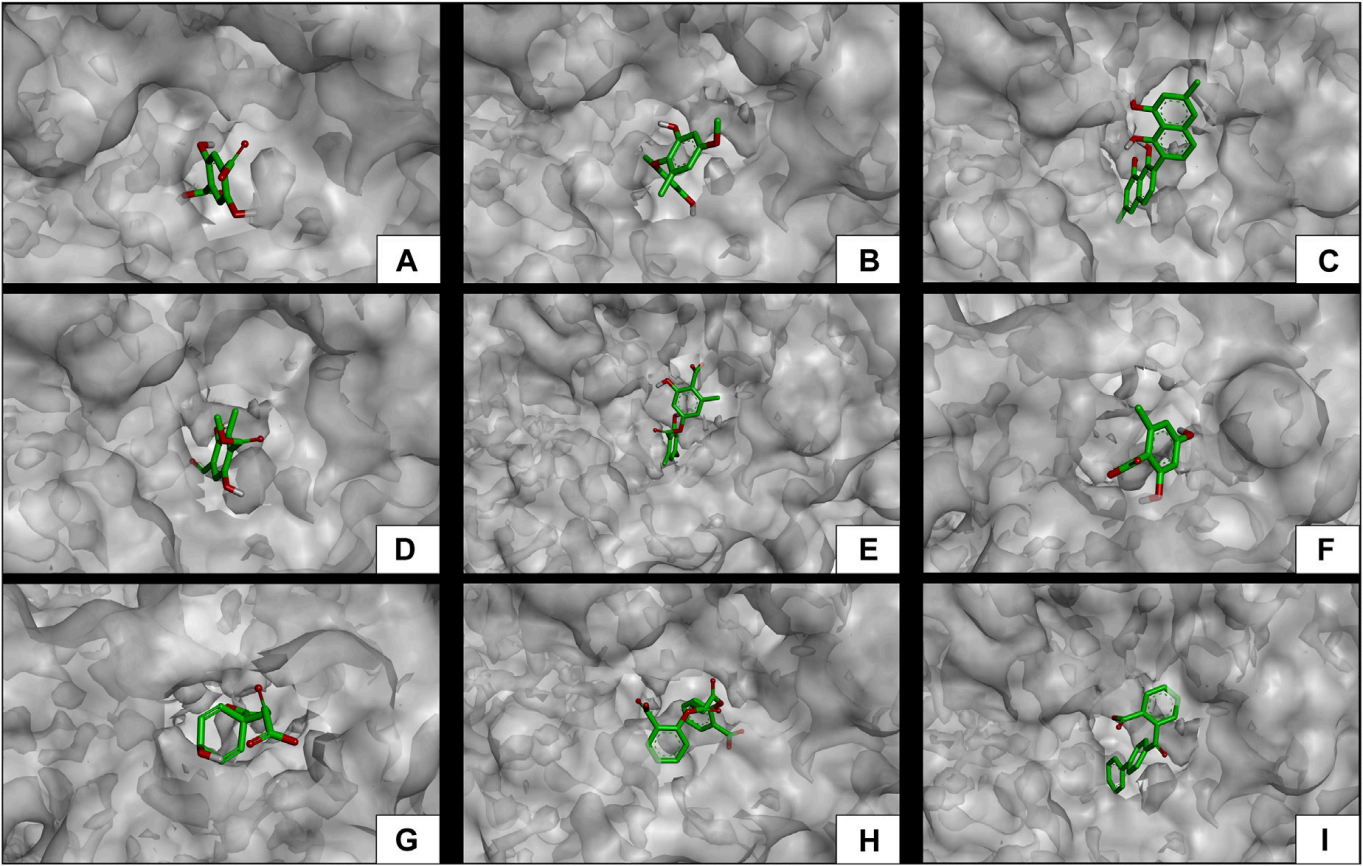
Docking molecular between phytochemicals and the *α*-glucosidase enzyme in a surface view. **(A)** *α*-Glucosidase and 2,5DHA; **(B)** *α*-glucosidase and cyperine; **(C)** *α*-glucosidase and diospyrol; **(D)** *α*-glucosidase and hypoxyphenone; **(E)** *α*-glucosidase and lecanoric acid; **(F)** *α*-glucosidase and orsellinic acid; **(G)** *α*-glucosidase and prephenic acid; **(H)** *α*-glucosidase and SDA; and **(I)** *α*-glucosidase and O4BBA.

**FIGURE 8:**
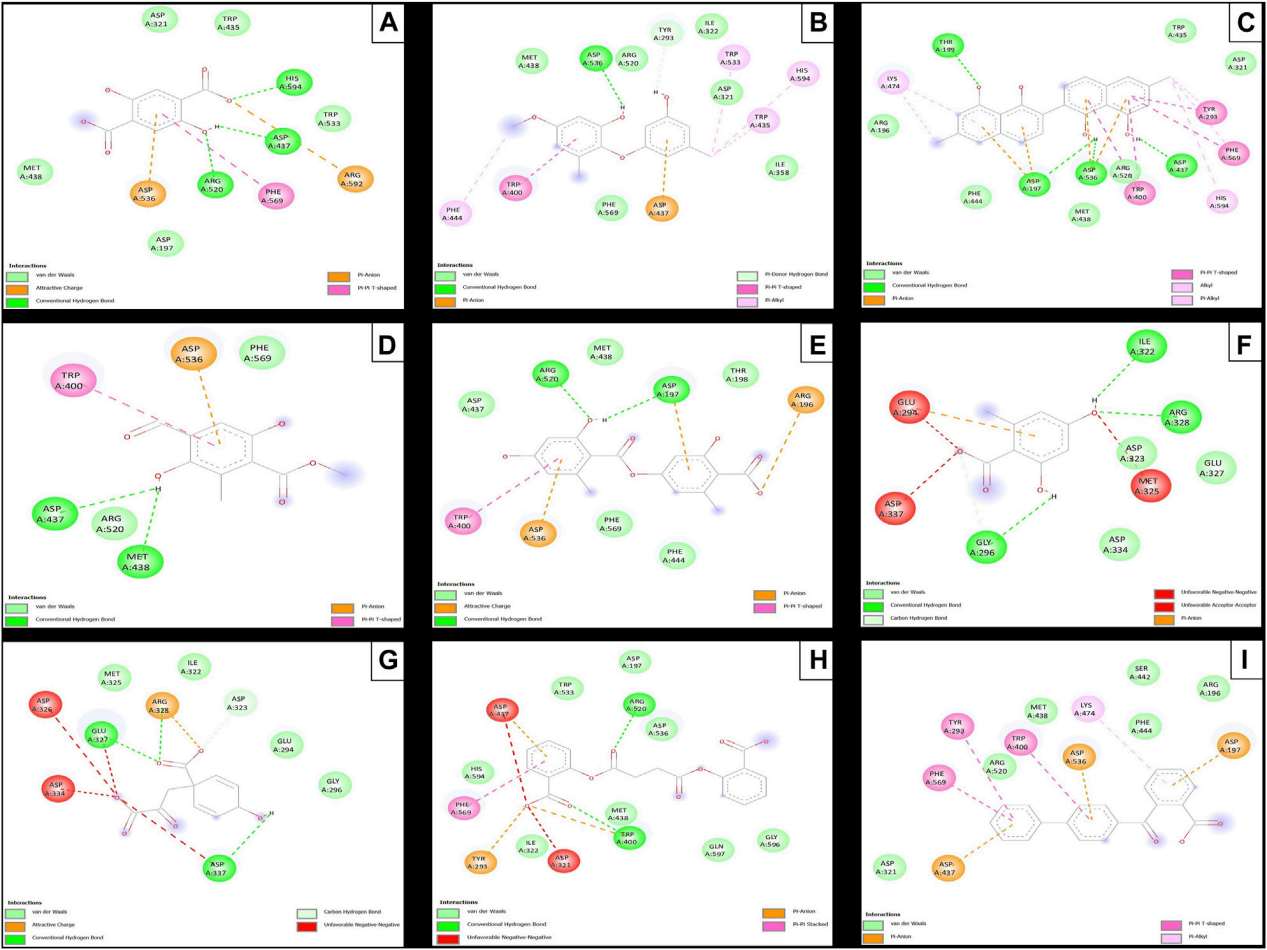
Molecular interactions between phytochemicals and the α-glucosidase enzyme. **(A)** Molecular interactions between 2,5DHA and *α*-glucosidase; **(B)** molecular interactions between cyperine and *α*-glucosidase; **(C)** molecular interactions between diospyrol and *α*-glucosidase; **(D)** molecular interactions between hypoxyphenone and *α*-glucosidase; **(E)** molecular interactions between lecanoric acid and *α*-glucosidase; **(F)** molecular interactions between orsellinic and acid *α*-glucosidase; **(G)** molecular interactions between prephenic acid and *α*-glucosidase; **(H)** molecular interactions between SDA and *α*-glucosidase; and **(I)** molecular interactions between O4BBA and *α*-glucosidase.

The results of the *in silico* analysis of the phytochemicals and *α*-glucosidase enzyme are shown in Figures 7, 8. The compounds were compared with the reference inhibitor acarbose to see if the behavior of the phytochemicals was similar or better against enzyme α-glucosidase. For each of the compounds, together with acarbose, their binding affinity (Table 9) against the α-glucosidase enzyme was calculated. It was observed that the compounds diospyrol and O4BBA presented higher binding affinities (−8.80 and −8.40 kcal/mol, respectively) compared to the reference inhibitor acarbose (−7.00 kcal/mol) (Table 9). In the interaction map (Figure 8), it is observed that the diospyrol compound presented four H-bond-type interactions with the amino acids Asp197, Thr199, Asp437, and Asp536 (Figure 8C); in addition, this compound presented four *π*-anion-type interactions with the amino acids Asp197 and Asp536. These interactions presented by the compound diospyrol caused its affinity and geometry in the catalytic site to be more stable compared to the inhibitor acarbose (Figures 7C, 8C).

The O4BBA compound presented three *π*-anion-type interactions between the *π* electrons of its aromatic rings and the amino acids Asp197, Asp437, and Asp536 (Figure 8I) which allow an electrostatic attraction at the catalytic site of *α*-glucosidase (Figure 7I). Five van der Waals-type interactions were also observed with the amino acids Arg196, Asp321, Met438, Ser442, Phe444, and Arg520. The compounds SDA, cyperine, and lecanoric acid presented binding affinities (−7.30, −7.20 and −7.00 kcal/mol; respectively) similar to the inhibitor acarbose (−7.00 kcal/mol). The SDA compound presented two H-bond interactions, mainly with the oxygens of the carbonyl groups and the amino acids Trp400 and Arg520 (Figure 8H). Two π-anion-type interactions were presented with the amino acids Trp400 and Tyr293. However, it was observed that the SDA compound presented two unfavorable negative–negative interactions with the amino acids Asp321 and Asp437; these interactions directly affect the energetic and geometric stability within the catalytic site of α-glucosidase (Figure 7H).

Compounds showing lower binding affinities than acarbose were 2.5DHA, hypoxyphenone, orsellinic acid, and prephenic acid (−5.80, −4.90, −5.10l, and −4.90 kcal/mol, respectively). Of the compounds that presented lower affinities than acarbose, the one that presented important interactions in the catalytic site of α-glucosidase was 2.5DHA (Figures 7A, 8A). Three H-bond interactions were observed with the amino acids Asp437, Arg520, and His594 (Figure 8A). Two *π*-anion-type interactions were also observed between the *π* electrons of the aromatic ring and the amino acid Asp535 and the carboxylate with the amino acid Arg592.

### 3.7 Evaluation of docking pancreatic lipase inhibition

**FIGURE 9:**
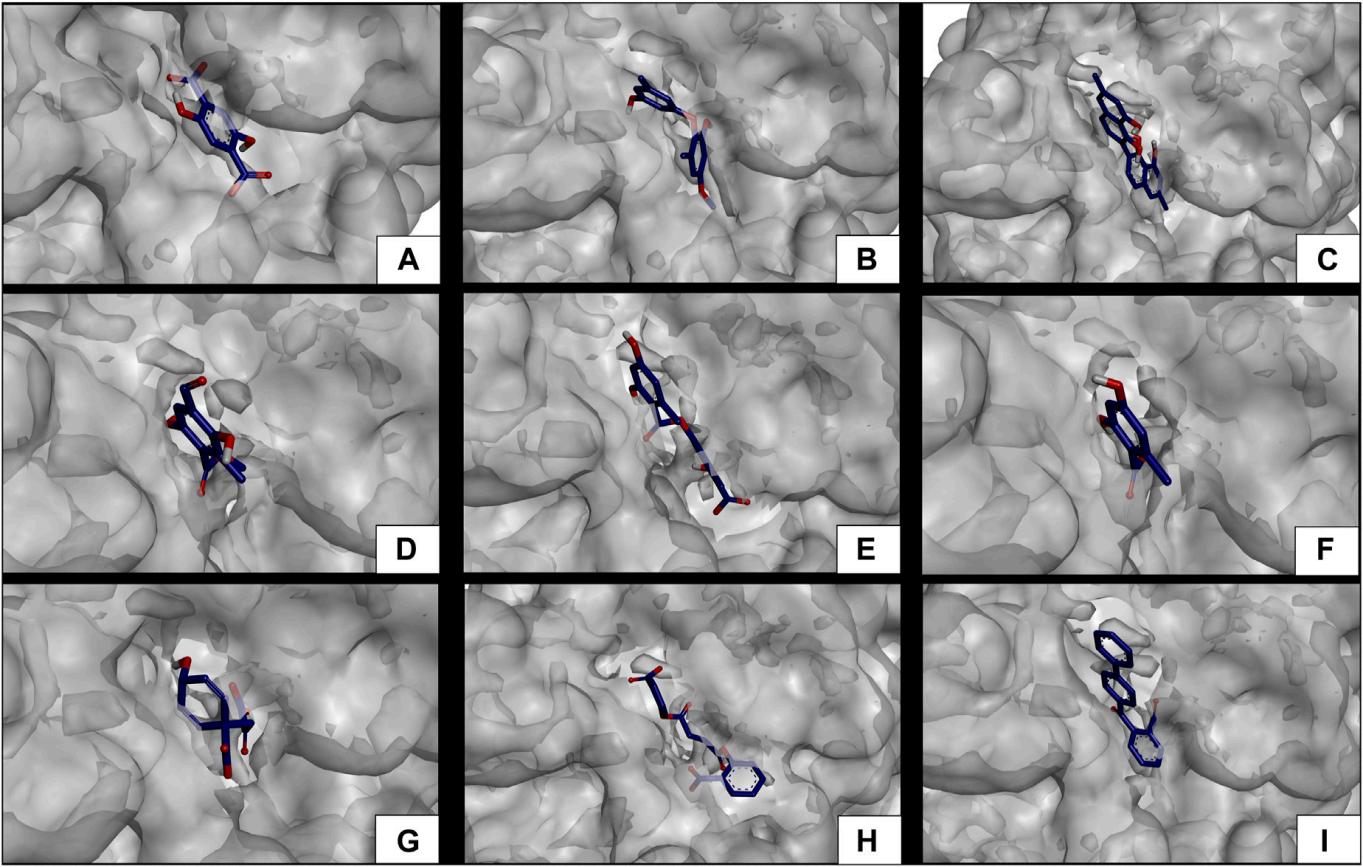
Docking molecular between phytochemicals and the human pancreatic lipase enzyme in a surface view. **(A)** Human pancreatic lipase and 2,5DHA; **(B)** human pancreatic lipase and cyperine; **(C)** human pancreatic lipase and diospyrol; **(D)** human pancreatic lipase and hypoxyphenone; **(E)** human pancreatic lipase and lecanoric acid; **(F)** human pancreatic lipase and orsellinic acid; **(G)** human pancreatic lipase and prephenic acid; **(H)** human pancreatic lipase and SDA; and **(I)** human pancreatic lipase and O4BBA.

**FIGURE 10:**
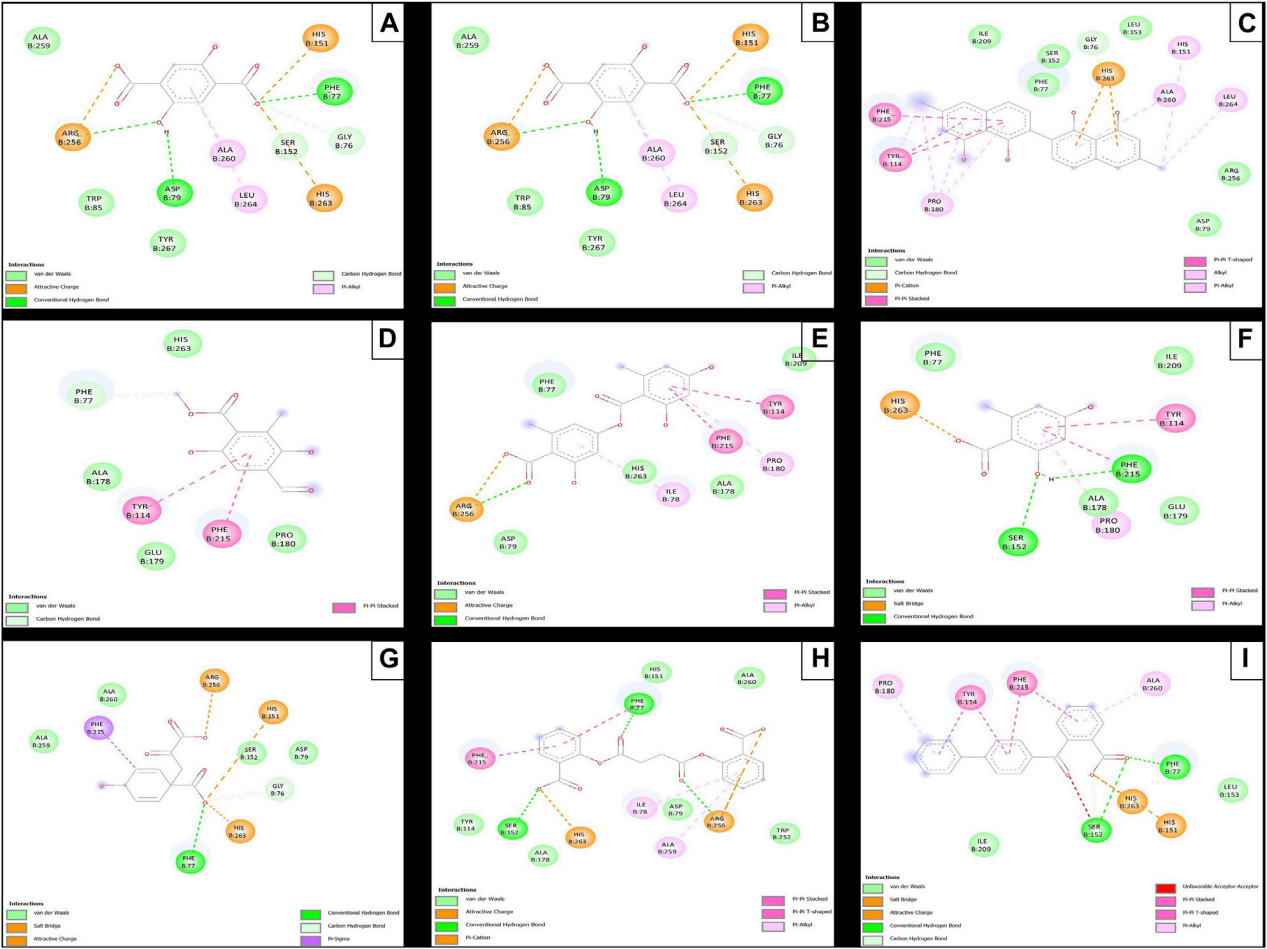
Molecular interactions between phytochemicals and the human pancreatic lipase enzyme. **(A)** Molecular interactions between 2,5DHA and human pancreatic lipase; **(B)** cyperine and human pancreatic lipase; **(C)** diospyrol and human pancreatic lipase; **(D)** hypoxyphenone and human pancreatic lipase; **(E)** lecanoric acid and human pancreatic lipase; **(F)** orsellinic and human pancreatic lipase; **(G)** prephenic acid and human pancreatic lipase; **(H)** SDA and human pancreatic lipase; and **(I)** O4BBA and human pancreatic lipase.

To carry out the *in silico* analysis of the phytochemicals, the cocrystallized ligand (MUP) of the human pancreatic lipase enzyme was used, which was used as a reference compound for coupling and to determine the coordinates of the catalytic site of the enzyme to the compound orlistat as a reference inhibitor since it is the only drug approved by the FDA that acts on pancreatic lipase. The binding affinity results showed that the compounds cyperine, diospyrol, lecanoric acid, SDA, and O4BBA presented binding affinities (−8.10; −11.0; −9.00; −8.50, and −10.5 kcal/mol, respectively) higher than those of the reference inhibitors orlistat and MUP (−7.10 and −5.70 kcal/mol, respectively). This behavior is mainly because the geometry adopted by these compounds in the catalytic site of the enzyme (Figure 9) allowed a better interaction with the amino acids directly involved in the catalytic sites (Ser152, Phe215, Arg256, His263, and Leu264). The diospyrol compound was the one that presented the highest binding affinity in the catalytic site (Figure 9C) because it presented two *π*-cation-type interactions between the *π* electrons of the aromatic ring and the amino acid His263, which is directly involved in the inhibition of human pancreatic lipase (Figure 10C).

In addition, five van der Waals interactions were observed with the amino acids Phe77, Asp79, Ser152, Leu153, Ile209, and Arg256 of which the amino acids Ser152 and Arg256 are directly involved in the enzyme binding site. The compound O4BBA was the second compound that presented a higher binding affinity in the human pancreatic lipase enzyme due to the conformation it adopted in the catalytic site (Figure 9I). This compound presented two H-bond-type interactions with the amino acids Phe77 and Ser152; however, it also presented an unfavorable acceptor–acceptor interaction with the amino acid Ser152, causing it to have an interaction similar to that of the diospyrol compound (Figure 10I). The compounds cyperine, lecanoric acid, and SDA showed similar conformations and binding affinities at the catalytic site of human pancreatic lipase (Figures 9, 10B, E, H). This behavior is mainly because they presented similar interactions with the residues directly involved in the catalytic site (Figure 10). The cyperine compound presented three H-bond-type interactions with the amino acids Phe77, Asp79, and Arg256; in addition, it presented attractive charge-type interactions between two carboxylate groups and a hydroxyl with the amino acids His151, His263, and Arg256 (Figure 10B). These interactions allowed adequate stabilization at the binding site. Figure 10E shows the main interactions presented by the lecanoric acid compound against the human pancreatic lipase enzyme. This compound presented some H-bond interactions with the amino acid Arg256 and five van der Waals-type interactions with the amino acids Phe77, Asp79, Ala178, Ile209, and His263 (Figure 10E).

The SDA compound presented three H-bond interactions with the amino acids Phe77, Ser152, and Arg256 (Figure 10H). It also presented two attractive charge-type interactions with the amino acids Arg256 and His263. These interactions with the amino acids that were in the binding site allowed the conformation of the SDA compound (Figure 10H) to stabilize and have better binding affinity with the reference inhibitors (orlistat and MUP).

According to molecular docking analyses, there is a greater potential for pharmacological effect when evaluating isolated compounds present in the extracts of *Psoroma* species, which is evident with other Antarctic lichen species, such as *H. lugubris* where the isolation of compounds such as usnic acid, barbatolic acid, atranol, and 5,7-dihydroxy-6-methylphthalide demonstrates greater antioxidant and enzyme inhibition activity (Areche et al., 2022); likewise, computational studies carried out with compounds present in the extracts of other Antarctic species, such as *L. brialmontii*, *P. pubescens*, *S. globosus*, *C. gracilis*, and *C. chlorophaea* (Torres-Benítez et al., 2022; Torres-Benítez et al., 2023a), validate the promising use of compounds of phenolic nature for the treatment of different pathologies of wide prevalence and incidence as well as the understanding of their mechanisms of action at the organismic and cellular level (White et al., 2014). In addition, in the last 2 decades, biologically active metabolites have been isolated, such as atranorin (Melo et al., 2011; Zhou et al., 2017; Urbanska et al., 2022), barbatic acid (Reddy et al., 2019), diffractaic acid (Karagoz et al., 2015; Emsen et al., 2018), evernic acid (Fernandez-Moriano et al., 2017a; Shameera Ahamed et al., 2019; Lee eta al., 2021), fumarprotocetraric acid (Kosanić et al., 2014; Fernández-Moriano et al., 2017b), gyrophoric acid (Candan et al., 2006; Bačkorová et al., 2011), lobaric acid (Hong et al., 2018), physodic acid (Studzińska-Sroka et al., 2021b), protocetraric acid (Nguyen et al., 2023), thamnolan (Omarsdottir et al., 2007), usnic acid (Luzina and Salakhutdinov, 2018), and vulpinic acid (Varol et al., 2016; Kılıç et al., 2018), which are present in the species of the genera *Cladonia, Parmotrema, Lepraria, Lethariella, Pseudoevernia, Hypotrachyna, Umbilicaria*, *Usnea*, among others.

## 4 Conclusion

The extracts of the lichenized fungi species *O. frigida*, *P. contortuplicata*, and *U. antarctica* contain various bioactive compounds of aromatic, carbohydrate, acid, lipid, and depside types that determine the possibility of positive effects on different biological activities *in vitro* and *in vivo*. The three species reported a moderately significant antioxidant activity that is proportionally related to the concentration of total phenols, especially more abundant in the lichen *O. frigida*. Regarding the *in vitro* enzyme inhibition activity, the extracts of the three species presented considerably better values for the *α*-glucosidase enzyme compared to the standard and less effective results for α-amylase and pancreatic lipase enzymes; however, with the *in silico* evaluation of a strong intermolecular interaction of compounds 2,5-dihidroxyterephthalic acid, cyperine, diospyrol, hypoxyphenone, lecanoric acid, orsellinic acid, prephenic acid, succinyldisalicylic acid, and o-(4-biphenylylcarbonyl) benzoic acid, the catalytic sites of *α*-glucosidase, *α*-amylase, and pancreatic lipase enzymes were detected. In this research, the potential of extracts and compounds of these species for the promising treatment of metabolic diseases such as diabetes mellitus and those related to oxidative damage such as Alzheimer’s, Parkinson’s and amyotrophic lateral sclerosis is highlighted. Furthermore, the prospects for the near future in this type of research is the biodirected study of ethanolic extract fractions, the isolation, elucidation, and biological assays of major compounds, and the testing of extracts, active fractions, and compounds in murine models with the aim of further strengthening the pharmacological, nutritional, and biomedical potential of the chemical world of lichenized mushrooms.

## Data Availability

The datasets presented in this study can be found in online repositories. The name of the repository and accession number can be found at: MetaboLights—MTBLS8292.
